# IgM specific to lipopolysaccharide of *Vibrio cholerae* is a surrogate antibody isotype responsible for serum vibriocidal activity

**DOI:** 10.1371/journal.pone.0213507

**Published:** 2019-03-07

**Authors:** Jae Seung Yang, So Jung An, Mi Seon Jang, Manki Song, Seung Hyun Han

**Affiliations:** 1 Clinical Research Laboratory, International Vaccine Institute, Seoul, Republic of Korea; 2 Vaccine Process Development, International Vaccine Institute, Seoul, Republic of Korea; 3 Department of Oral Microbiology and Immunology, DRI, and BK21 Plus Program, School of Dentistry, Seoul National University, Seoul, Republic of Korea; New York State Department of Health, UNITED STATES

## Abstract

Serum vibriocidal antibody assays have long been used to evaluate the immunogenicity of cholera vaccines formulated with killed whole-cell *Vibrio cholerae*. However, the antibody isotypes responsible for the serum vibriocidal activity are not fully characterized. In this study, we examined 20 clinical serum samples obtained from human subjects who had been vaccinated with a killed, whole-cell cholera vaccine and a positive control, human convalescent sera with high vibriocidal activity, to determine which isotype antibody is associated with the vibriocidal activity. Antibody isotypes from pooled convalescent sera were fractionated by size-exclusion column chromatography, and the major vibriocidal activity was detected in the IgM fraction. Depletion of IgM antibodies in the convalescent sera produced a significant (*P*<0.05) decrease in vibriocidal activity (16-fold decrease), whereas only a small change was observed with depletion of IgG or IgA. In addition, anti-LPS IgM antibody showed the highest correlation with vibriocidal activity (Spearman correlation coefficient *r* = 0.846) among antibody isotypes against heat-killed *V*. *cholerae*, lipopolysaccharide (LPS), or major outer membrane protein (Omp U), while total IgG, IgA, or IgM antibody level was not correlated with vibriocidal activity in the 20 human clinical serum samples. Furthermore, human convalescent sera significantly (*P*<0.001) inhibited the attachment of *V*. *cholerae* to HT-29, a human intestinal epithelial cell *in vitro*. Interestingly, IgM-depleted convalescent sera could not effectively inhibit bacterial adherence compared with non-depleted sera (*P*<0.05). Finally, bacterial adhesion was significantly inhibited by sera with high vibriocidal titer compared with low-titer sera (*P* = 0.014). Collectively, we demonstrated that anti-*V*. *cholerae* LPS IgM is highly correlated with serum vibriocidal activity and it could be a surrogate antibody isotype representing protective antibodies against *V*. *cholerae*.

## Introduction

*Vibrio cholerae* causes acute diarrhea by cholera toxin-mediated intestinal fluid secretion in humans. Humoral immunity rather than cellular immunity has been considered to play a role in protection against cholera because the bacteria do not invade the intestinal epithelial barrier and are unable to survive intracellularly [1]. Humoral immunity against *V*. *cholerae* has long been focused on *V*. *cholerae* LPS for protective immunity due to its association with protection in humans [1]. In addition, mucosal and systemic humoral responses against outer membrane protein [2], cholera toxin B subunit [3], and toxin-coregulated pilus (TCP) [4] have also been induced in cholera patients.

Although specific antibodies against *V*. *cholerae* are known to play a role in protection in humans, there is no direct evidence as to which antibody isotype is the most important to provide immunity against cholera. Two isotypes, secretory IgA and serum IgG, have been shown to provide protective immunity against *V*. *cholerae* infection [5, 6], and LPS-specific IgA is increased in the sera and intestinal fluids from patients and vaccinated individuals [7–9]. Significant levels of *V*. *cholerae* LPS-specific serum IgG are also augmented in response to *V*. *cholerae* infection [10–12].

Given that IgG and IgM can trigger complement-mediated bacterial lysis through the classical complement pathway [1], IgM could be also responsible for protection against *V*. *cholerae*. It is conceivable that IgM is much superior to IgG in complement-fixing activity because its pentameric structure has higher functional avidity than monomeric IgG [13]. Single IgM binds several identical epitopes on bacterial surface and it allows globular heads of C1q initiating the complement activation, while multiple IgG molecules are required for binding of C1q [13]. In addition, high vibriocidal and agglutinating antibody titer were observed in both IgM and IgG fraction of adult cholera cases in endemic area [14]. Furthermore, LPS-specific IgM was increased in cholera patients [15], and higher levels of IgM than of IgA were found in gut lavage fluids from infants with acute diarrhea [16].

The vibriocidal assay is an *in vitro* test that measures the ability of antibodies mediated by complements to kill virulent *V*. *cholerae*. Vibriocidal antibody levels increase by aging and have been shown to be inversely associated with susceptibility to cholera in endemic area [17, 18]. Moreover, a previous study showed a good correlation between vibriocidal titer and protection against disease caused by *V*. *cholerae* O1 [3]. Therefore, serum vibriocidal antibody titer has been used as a representative marker of immunity to *V*. *cholerae*. In general, although there is no known threshold of vibriocidal titer for protection from the disease, a 4-fold increase or higher in vibriocidal titer after the vaccination has been widely accepted as an effective immunization [19]. For accurate assessment of cholera vaccine-induced immunity, it is important to identify antibody isotype responsible for vibriocidal activity. Understanding of immunological properties of protective antibody would facilitate to design more effective vaccines. In the present study, we investigated the antibody isotypes and specific antigen associated with serum vibriocidal function and their inhibitory activity against bacterial colonization on human intestinal epithelial cells.

## Materials and methods

### Bacteria and reagents

*V*. *cholerae* O1 El Tor Inaba (strain T19479) was kindly provided by Prof. Jan Holmgren (Gothenburg University, Sweden) and was used as the target bacteria for the vibriocidal assay. Brain Heart Infusion (BHI) media and Guinea pig complements were purchased from Difco (San Jose, CA, U.S.A.) and Rockland (Gilbertsville, PA, U.S.A.), respectively. Protein G-agarose (PGA) and rabbit anti-human IgM (μ-chain specific) or IgA (α-chain specific) in rabbit IgG fraction were purchased from Sigma-Aldrich Co. (St. Louis, MO, U.S.A.).

### Serum samples

Convalescent sera with high vibriocidal antibody titers of ≥ 8,000 were pooled from cholera patients in India and used to deplete target isotype of antibody. Bivalent oral cholera vaccine (Shanchol, Shantha Biotechnics), formulated with *V*. *cholerae* O1 Inaba, O1 Ogawa, and O139, was given with two doses at two week interval (days 0 and 14) through clinical trials [20–22]. Blood samples were obtained to use in this study from volunteers at 2 weeks (days 14 and 28) after first dose and second dose, respectively. Human sera were heated at 56°C for 30 min before use to inactivate complements. After approval from the ethics committee of the National Institute of Cholera and Enteric Diseases, the Health Ministry Screening Committee of India, and the Institutional Review Board of the International Vaccine Institute, all serum samples were used.

### Depletion of antibody isotypes

One milligram of PGA was initially mixed with 100 μg of IgG fraction of rabbit anti-human IgM (μ-chain specific) or IgA (α-chain specific) at room temperature for 1 h. Convalescent sera were then mixed with anti-human IgM- or IgA-bound PGA and followed by precipitation of the antibody-agarose complex to deplete serum IgM or IgA. PGA was used to deplete IgG in the convalescent sera. The supernatants were then incubated again with fresh PGA, anti-IgM-, or anti-IgA-bound-PGA; this clearance procedure was repeated 7 consecutive times.

### Purification of *V*. *cholerae* LPS and recombinant OmpU

LPS was purified from *V*. *cholerae* O1 Inaba (strain T19479) using LPS extraction kit (Intron, Seongnam, Korea) according to the manufacturer’s instruction as previously described [23]. The *ompU* gene was amplified from *V*. *cholerae* O1 Inaba (strain T19479) by PCR and recombinant OmpU was expressed in the *E*. *coli* BL21 (DE3), and purified as previously described [24].

### Quantification of antibody isotypes using ELISA

To quantify total antibody isotypes in serum samples, a 96-well plate (Nunc, Roskilde, Denmark) was coated with anti-human κ and λ light chain antibodies (Bethyl Laboratories, Montgomery, TX, USA) overnight at 4°C and blocked with BSA. Serially diluted sera and human IgM, IgG, or IgA (1 μg/ml) (Jackson Immunoresearch, West Grove, PA, USA) were added. After incubation at 37°C for 2 h, serum antibody isotypes were detected with alkaline phosphatase-conjugated anti-human IgM, IgG, or IgA (Jackson Immunoresearch) and developed with 4-nitrophenyl phosphate (Fluka Chemie, Buchs, Switzerland). Optical density was read at 405 nm using a microplate reader (Molecular Device, Sunnyvale, CA, USA). To measure *V*. *cholerae*-specific serum antibodies, plates were coated with *V*. *cholerae* LPS (2.5 μg/ml), heat-killed *V*. *cholerae* (HKVC) (10^8^ CFU/ml), or recombinant OmpU (rOmpU, 2.5 μg/ml) at 4°C overnight. Titers of total antibody and *V*. *cholerae*-specific antibody were calculated with Softmax Pro version 4.8 (Molecular Device). Antibody titers below 40 were considered as 20 for statistical analysis as described previously [25].

### Vibriocidal assay

The vibriocidal assay was performed as previously described (Yang et al., 2007). Briefly, serum samples were diluted with 0.85% saline from 1 in 2.5 to 1 in 1,280 or more as necessary. Each well of a 96-well plate (Nunc, Roskilde, Denmark) was filled with 25 μl of sample. Fresh colonies of *V*. *cholerae* O1 (strain T19479) were cultured in 20 ml of BHI media for 2–3 h at 37°C until they reached mid-log phase. Bacteria were collected by centrifugation, washed with saline, and diluted with saline containing 10% guinea pig complements to adjust to 1×10^6^ cells/ml. An equal volume of diluted bacteria was added to the 96-well plate containing serially-diluted serum samples, and the plates were incubated at 37°C for 1 h. Next, 150 μl of fresh BHI broth was added, and plates were incubated for an additional 4 h. Bacterial growth was measured at OD_600_ using a microtiter-plate reader (Spectramax 190, Molecular Device, Sunnyvale, CA, USA). The definition of vibriocidal antibody titer was the reciprocal of the highest serum-dilution fold completely inhibiting bacterial growth.

### Fractionation of convalescent sera

To see which isotype was most closely correlated with vibriocidal activity, convalescent sera were separated by size exclusion chromatography using Superdex 200 10/300GL (GE Healthcare, USA) as previously described [26]. A 200 μl diluted serum sample (1 in 10) was applied to the column and run at a flow rate 0.5 ml/min in 0.2 M NaCl (pH 7.0). Forty fractions (0.5 ml) were collected and used to examine the levels of total IgG, IgA, IgM, and vibriocidal titer.

### Bacterial adhesion assay

HT-29 cells, cultured in a 24-multi-well plate at 2 x 10^5^ cells per well at 37°C in an incubator with 5% CO_2_ for 1 week until the cells reached confluence, were used for adhesion assay as previously described [24]. Briefly, *V*. *cholerae* was grown at 37°C overnight in LB broth and washed three times with PBS. Then, the bacteria (1 x 10^6^ CFU/ml) were incubated with serially-diluted sera or specific isotype-depleted sera for 1 h at 4°C and added to a 24-well plate containing HT-29 cells. After 1 h incubation with gentle shaking, HT-29 cells were washed with PBS to remove unbound bacteria and lysed cells using 0.2% Triton X-100 for 10 min. The lysed cells were diluted and plated on LB agar plate to determine the number of adherent bacteria.

### Statistical analysis

To compare vibriocidal antibody titers and the levels of LPS-specific antibody isotypes, the results of each serum sample obtained by two assays were plotted against each other. The relationship between the two values was examined by Spearman correlation coefficient (*r*), and *P* values were obtained using GraphPad Prism 5 software (GraphPad Software, La Jolla, CA, USA). Statistical differences between two groups were determined by two-tailed Student’s t-tests.

## Results

### Vibriocidal activity profile was coincident with the serum IgM level

**Fig 1:**
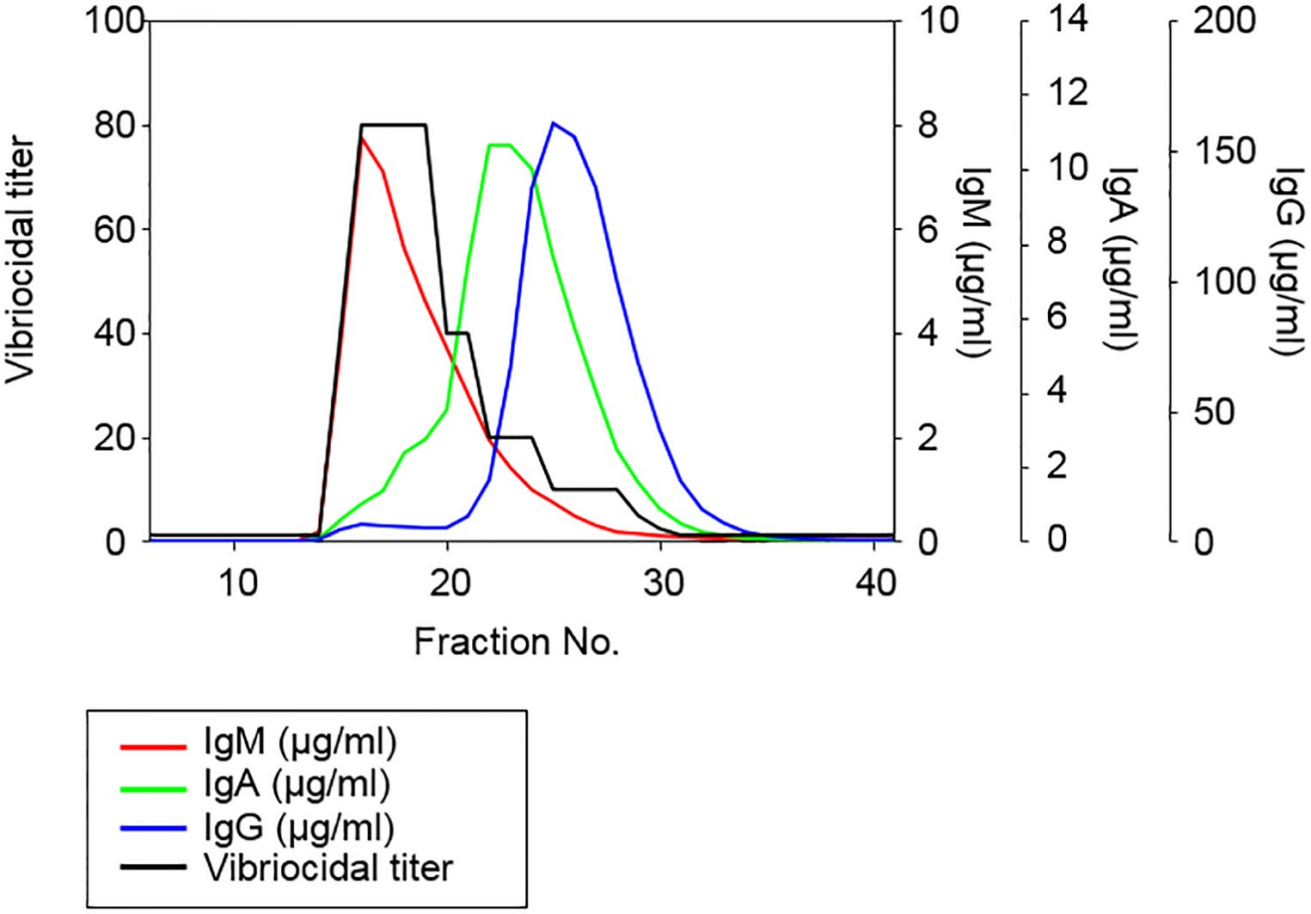
High vibriocidal activities were detected in the IgM fraction of the convalescent sera. Pooled convalescent sera were fractionated by size exclusion chromatography using Superdex 200 10/300GL. Vibriocidal titers and levels of total IgG, IgA, and IgM were determined in 40 fractionated serum samples.

To examine the correlations between antibody isotype and vibriocidal activities, pooled convalescent sera from cholera patients were fractionated using size exclusion chromatography. The vibriocidal activities and the total amounts of IgM, IgG, and IgA were measured in the 40 chronologically-obtained fractions. As shown in Fig 1, vibriocidal titers were measured from 2.5 to 80 in the fractions between No.15 and No. 30. The maximal vibriocidal titer was observed as 80 in the fraction between No. 16 and No. 19, and serum IgM was quantified as 7.7 μg/ml with a peak in the fraction No.16. Here, 10.6 μg/ml of IgA and 160.2 μg/ml of IgG reached their peaks in No. 22 and No. 25, and their vibriocidal titers were 20 and 10, respectively. Vibriocidal titers were decreased in parallel with the level of IgM but were not associated with the levels of IgG nor IgA, indicating that vibriocidal titers are dependent on IgM level in the serum.

### Depletion of IgM abrogated vibriocidal activity in serum

**Fig 2:**
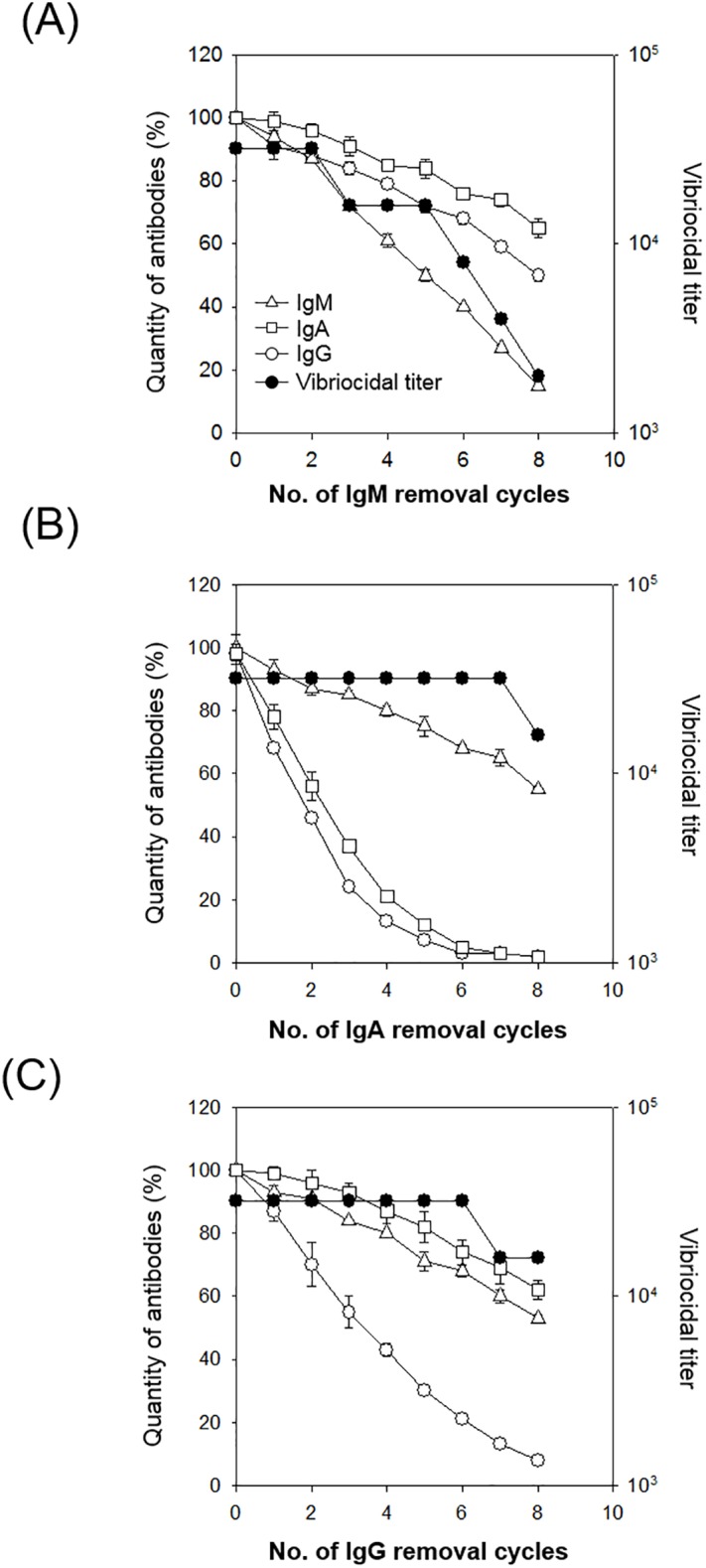
Vibriocidal activities were significantly decreased by depletion of IgM in serum. Pooled convalescent sera were incubated with anti-human IgM, anti-human IgA-bound protein G-agarose, or protein G-agarose to remove IgM (A), IgA (B), and IgG (C), respectively. The levels of antibody isotypes (Δ, IgM; □, IgA; ○, IgG) were determined using ELSIA and were compared with their vibriocidal activity (●) in the supernatant. Values are shown mean ± standard deviation of triplicate assays.

To confirm that serum IgM plays a critical role in vibriocidal activity, we depleted IgM in the convalescent sera and then measured the level of IgM and vibriocidal antibody titer. To deplete IgM, sera were mixed with anti-IgM antibody-bound PGA followed by precipitation of complex. As shown in Fig 2A, the amount of serum IgM was significantly decreased by repeating the depletion procedure, and only 15% of the original IgM remained. Concomitantly, vibriocidal antibody titers were also sequentially decreased from 32,000 to 2,000 by carrying out the IgM depletion process. Although the amount of serum IgG and IgA was also decreased, they still remained at high levels (*i*.*e*., 50% and 65% of initial levels) in the final step, respectively, during the IgM removal procedure despite about 16-fold decrease in the vibriocidal titers (Fig 2A). Serum IgG and IgA were also depleted by incubation with PGA or anti-IgA antibody-bound PGA, respectively, and we measured the amounts of antibody and vibriocidal antibody titers in sera (Fig 2B and 2C). Serum IgG and IgA levels were significantly diminished by repeating the removal step and finally remained at 8% and 2% of the initial levels, respectively. However, only a two-fold decrease in vibriocidal titer (from 32,000 to 16,000) was observed in the final step. Therefore, these results indicate that serum IgM might be a crucial isotype for vibriocidal activity.

### Vibriocidal titers were highly correlated with anti-*V*. *cholerae* LPS IgM level

Total serum IgG, IgA, and IgM levels were measured to determine correlation with vibriocidal antibody titers in 20 clinical sera from vaccinees given the cholera vaccine (Table 1). Twenty serum samples were shown to have various amounts of isotypes in ranges from 7.97 to 15.88 mg/ml for IgG, 0.49 to 2.18 mg/ml for IgA, and 0.34 to 1.86 mg/ml for IgM. No correlation was found between total amount of each isotype and vibriocidal titer of clinical sera.

**Table 1:** Total amount of antibodies and vibriocidal titers in the clinical sera.

Arbitrary No.	Antibody (mg/ml)			Vibriocidal titer
	IgM	IgG	IgA	
1	0.63	15.56	1.45	640
2	0.60	14.13	0.52	640
3	1.50	15.88	1.82	2560
4	0.98	11.65	2.18	2560
5	0.55	15.68	1.53	2560
6	0.61	14.29	0.63	640
7	1.86	10.97	0.62	5120
8	0.57	12.59	0.49	5120
9	1.55	7.97	0.62	5120
10	0.34	14.31	1.16	5120
11	0.54	15.90	1.04	5120
12	0.77	12.54	0.82	1280
13	0.62	13.25	0.72	5120
14	0.62	9.59	1.37	5120
15	1.05	11.94	0.62	5120
16	0.69	12.68	1.61	5120
17	0.76	10.11	1.31	20480
18	0.65	11.40	1.50	20480
19	0.54	12.90	1.39	10240
20	0.62	10.81	1.62	10240

**Fig 3:**
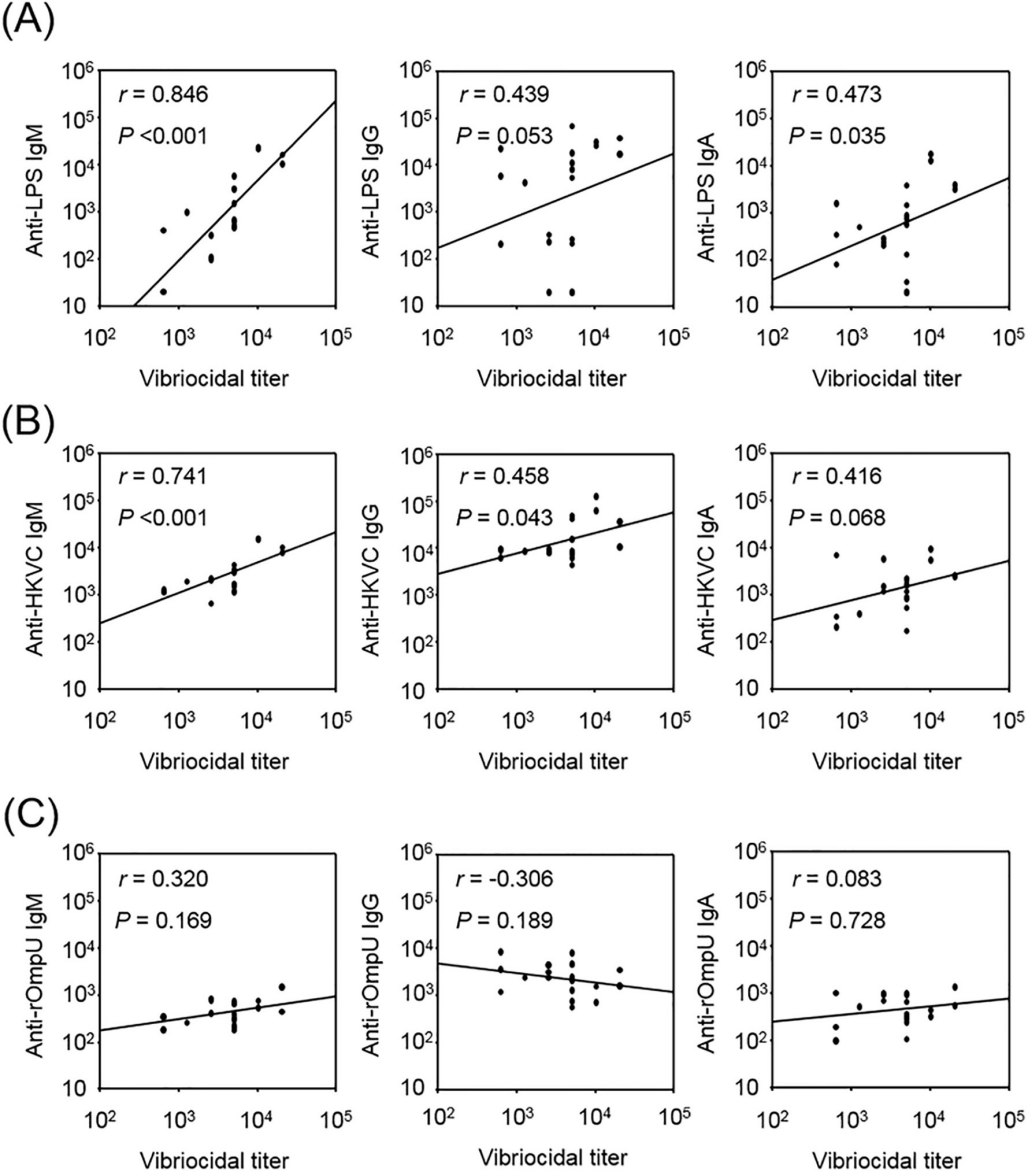
Vibriocidal titer was highly correlated with anti-*V*. *cholerae* LPS IgM level. The levels of serum IgM, IgG, and IgA against *V*. *cholerae* LPS (A), heat-killed *V*. *cholerae* (HKVC) (B), and *V*. *cholerae* OmpU (C) in 20 clinical serum samples were determined using ELISA and compared with their vibriocidal activities. Diagonal lines indicate regression between the two results. The Spearman correlation coefficient (*r*) and P value were obtained to compare the two results.

Next, we quantified antibody isotypes against heat-killed *V*. *cholerae* (HKVC), LPS, or recombinant outer-membrane protein U (rOmpU), which is the most abundant outer-membrane protein of *V*. *cholreae*, to see whether there was a correlation between *V*. *cholerae*-specific antibody isotype and vibriocidal titer. As shown in Fig 3, the levels of LPS-specific and HKVC-specific serum IgM were significantly correlated with vibriocidal titer. Statistical analyses demonstrated that the Spearman correlation coefficient (*r*) between IgM and vibriocidal titer was 0.846 (*P* < 0.001) against *V*. *cholerae* LPS, 0.741 (*P* < 0.001) against HKVC, and 0.320 (*P* = 0.169) against OmpU. In contrast, serum vibriocidal titer was shown to be more weakly correlated with *V*. *cholerae*-specific IgA or IgG; *r* = 0.439 (*P* = 0.053) for anti-LPS IgG, *r* = 0.473 (*P* = 0.035) for anti-LPS IgA, *r* = 0.458 (*P* = 0.043) for anti-HKVC IgG, *r* = 0.416 (*P* = 0.068) for anti-HKVC IgA, *r* = -0.306 (*P* = 0.189) for anti-OmpU IgG, and *r* = 0.083 (*P* = 0.728) for anti-OmpU IgA. These results imply that serum anti-LPS IgM is most highly correlated with vibriocidal antibody.

### Clinical sera with high vibriocidal titer significantly inhibited bacterial adhesion to intestinal epithelial cells

**Fig 4:**
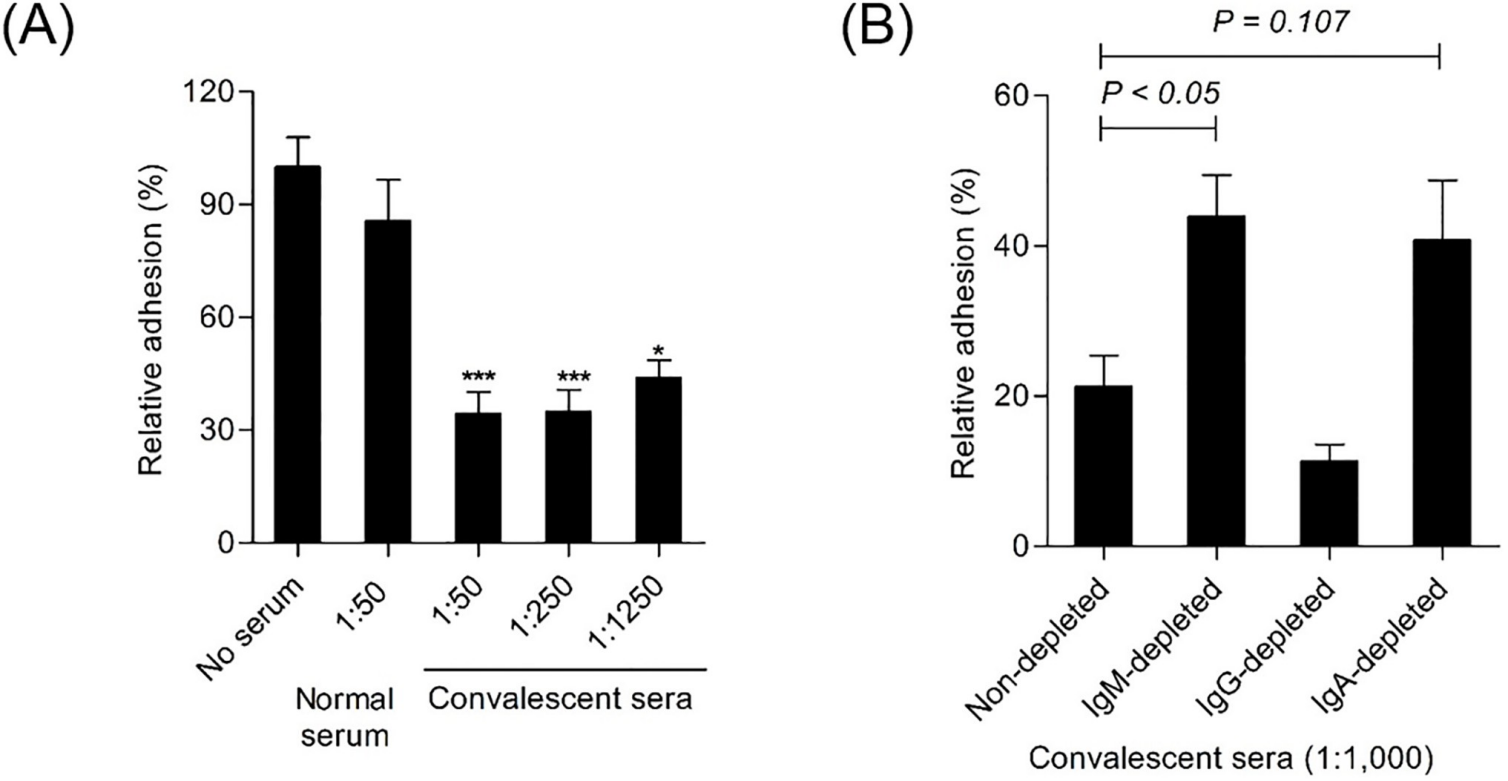
Convalescent sera, but not IgM-depleted sera, inhibited bacterial adhesion to intestinal epithelial cells. HT-29 cells (2 × 10^5^ cells/ml) were cultured in a 24-well plate for 8 days until cells reached confluence. *V*. *cholerae* O1 Inaba (1 x 10^6^ CFU/ml) was incubated with serially diluted convalescent sera (A) or antibody isotypes-depleted sera (B) for 1 h at 4°C and added to HT-29 cells. After additional 1 h incubation at room temperature with gentle shaking, HT-29 cells were washed with PBS followed by treatment with 0.2% TritonX 100. Adherent bacteria were cultured overnight on LB agar plates and counted. Values are mean ± standard deviation of triplicate samples.

Since *V*. *cholerae* colonizes to the intestinal epithelial layer to cause disease [27], interference of interactions between the pathogen and host cells at early stage is essential for prevention of disease. To investigate a role of anti-*V*. *cholerae* sera in the inhibition of bacterial adherence, bacteria pre-incubated with various dilutions of human convalescent sera and washed were applied to HT-29 human intestinal epithelial cells. As shown in Fig 4A, normal serum obtained from a placebo did not affect the binding ability of *V*. *cholerae* to HT-29 cells, but all diluted convalescent sera significantly inhibited bacterial adhesion compared with the control group (*P* < 0.001). Next, to see which isotype(s) of antibodies is(are) important for inhibition of bacterial adherence, *V*. *cholerae* was mixed with each isotype-depleted convalescent serum for 1 h at 4°C and applied to HT-29 cells (Fig 4B). Non-depleted and IgG-depleted sera significantly blocked bacterial adhesion to 21% and 11%, respectively, while bacterial adherence was significantly restored up to 44% and 41% when *V*. *cholerae* was pre-treated with IgM-depleted sera (*P* < 0.05) and IgA-depleted sera (*P* = 0.107), respectively. These data indicate that human anti-*V*. *cholerae* antibodies play a pivotal role in prevention of the bacterial colonization in the human epithelial layer. Our results suggest that IgM is the isotype responsible for inhibition of bacterial adherence, with IgA playing a less important role.

**Fig 5:**
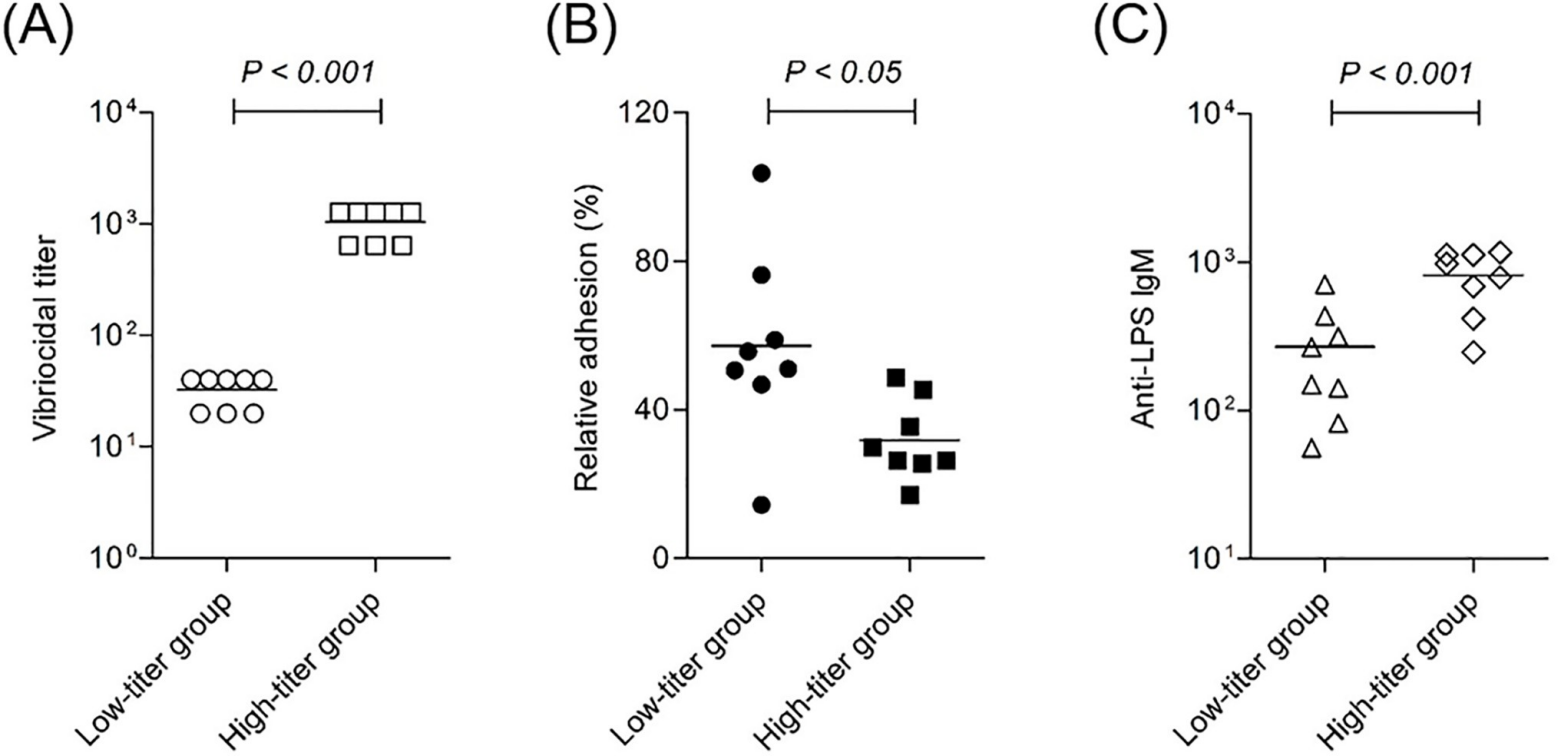
Clinical sera with high vibriocidal titer had a large amount of anti-*V*. *cholerae* LPS IgM and significantly inhibited bacterial adhesion to HT-29 cells. Sixteen sera containing low or high vibriocidal antibody (A) were examined for inhibitory activity of bacterial adhesion to HT-29 cells (B) and anti-*V*. *cholerae* LPS IgM level (C). To see the effect of clinical sera on bacterial adhesion to intestinal epithelial cells, HT-29 cells (2 × 10^5^ cells/ml) were cultured in a 24-well plate for 8 days until cells reached confluence. *V*. *cholerae* O1 Inaba (10^6^ CFU/ml) were pre-incubated with each clinical sera with low or high vibriocidal activity for 1 h, added to HT-29 cells, and then incubated for 1 h. HT-29 cells were washed with PBS and detached with 0.2% TritonX 100. Adherent bacteria were cultured overnight on LB agar plates and counted. Results are plotted as average of triplicates.

To further confirm whether serum vibriocidal activities have a correlation with bacterial adhesion, 16 human sera with high and low vibriocidal titers were selected from clinical samples and divided into low- and high-titer groups. As shown in Fig 5A, vibriocidal titers were 20 or 40 in the low-titer group and 640 or 1280 in the high-titer group. Adhesion of *V*. *cholerae* to differentiated HT-29 cells was significantly (*P <* 0.05) inhibited by pre-treatment with high-titer sera compared with pre-treatment with low-titer sera (Fig 5B). Interestingly, the geometric mean titer of anti-LPS IgM for the high-titer group was significantly (*P*< 0.001) higher at 729 (range from 417 to 1163) than that for the low-titer group at 199 (range from 56 to 433) (Fig 5C). Considering that vibriocidal titers are inversely correlated with bacterial adhesion and positively associated with the amount of IgM against *V*. *cholerae* LPS, this antibody isotype in serum would be an indirect functional marker for prevention of bacterial attachment in the intestinal mucosa.

## Discussion

Serum vibriocidal antibody titer has been widely used to evaluate protective immunity to cholera in populations suffering from infection or in those administered cholera vaccines [28, 29]. Previous reports have indicated that serum IgG is an important isotype for protection against cholera [6, 30]. However, the serum antibody isotypes responsible for vibriocidal antibody activity are not fully understood. In the present study, we found that total IgG, IgA, or IgM antibody level was not associated with vibriocidal titer, but *V*. *cholerae* LPS-specific IgM was highly correlated with its bactericidal activity in the clinical specimen. The correlation was further demonstrated by showing that the fractions responsible for vibriocidal activity were IgM rather than IgG or IgA, and that depletion of IgM showed significant decrease in vibriocidal titer, which was not shown with either IgG or IgA depletion. In addition, sera with high vibriocidal titer and anti-*V*. *cholerae* LPS IgM level substantially inhibited the attachment of *V*. *cholerae* to intestinal epithelial cells, while sera with low vibriocidal titer or IgM-depleted sera did not. All these data indicate that serum anti-*V*. *cholerae* LPS IgM is responsible for vibriocidal activity and inhibition of bacterial adhesion to intestinal mucosa.

Our results suggest that anti-*V*. *cholerae* LPS IgM is responsible for vibriocidal activity in serum, which was also found in other studies on *V*. *cholerae* as well as other bacterial infectious diseases [11, 14, 31, 32]. For example, serum IgM against pneumococcal capsular polysaccharide showed higher correlation with opsonophagocytic activity than IgG in individuals immunized with pneumococcal conjugate vaccine [32]. In addition, lack of serum bactericidal activity against *Escherichia coli* in patients with liver cirrhosis was also associated with diminished IgM [33]. In another study, purified IgG and IgM from sera of adults immunized with *Haemophilus influenzae* Type b conjugate vaccine showed bactericidal activity and protective immunity in an infant rat model [31]. However, IgG1 and IgG3, but not IgM, are responsible for protection against meningococcal infection and are positively correlated with bactericidal activity [34, 35]. In cholera, it has been also reported that *V*. *cholerae* LPS-specific IgG1, IgG3, and IgM are important for vibriocidal activity and agglutination antibody [11, 14]. In particular, IgM is a major isotype responsible for the vibriocidal activity after the primary exposure to *V*. *cholerae* or the vaccination. On the other hand, IgG1 and IgG3 are also associated with vibriocidal antibody as well as IgM in the secondary exposure [11, 14]. Recently, IgM against O-specific polysaccharide of LPS may specifically mediate vibriocidal antibody responses in the vaccinee administered with oral cholera vaccine [36]. Thus, IgM and/or IgG tend to have a positive correlation with serum bactericidal activity, and the bactericidal antibody isotype may differ by vaccine.

*V*. *cholerae* LPS is considered as a major virulence factor, and anti-*V*. *cholerae* LPS antibody is closely associated with protective immunity against cholera [37, 38]. In fact, oral cholera vaccination effectively elicited serum IgG, IgM, and IgA antibody responses against LPS or OSP while magnitude of antibody levels between IgG and IgM was similar in adults [39] and children [36]. It is consistent with our results that geometric mean titer of anti-LPS IgG and IgM was not significantly different (*data not shown*). The role of LPS was further supported by a previous result that cross-serotype challenge of *V*. *cholerae* O1 after primary exposure to either O1 Inaba or O1 Ogawa in human volunteers elicited prolonged stool excretion of vibrio compared with homologous serotype challenge [11]. In the present study, we first demonstrated that elevated anti-*V*. *cholerae* LPS IgM and vibriocidal antibody titer after cholera vaccination have a positive correlation with inhibition of bacterial colonization in human epithelial cells. Similar results have been reported previously, indicating that vibriocidal antibody, but neither anti-*V*. *cholerae* LPS IgG nor anti-toxin IgA, is associated with protection against *V*. *cholerae* O1 colonization and disease occurrence [3]. Notably, vibriocidal antibody has been detected in the bile fluid [40], and a 10-fold increase of IgM was reported in the intestinal fluid from children with acute diarrheal disease [16]. Previous studies have shown that *V*. *cholerae* O1 adheres to M cells in the human small intestine and is transported to Peyer’s patches for induction of immune responses [41, 42]. Fewer *V*. *cholerae* O1 cells were attached to M cells in immunized rats compared with unimmunized animals, suggesting that *V*. *cholerae* infection interferes with active mucosal immune responses [41]. Anti-*V*. *cholerae* LPS IgM may play a role in the inhibition of bacterial adherence.

Here, we found that bacterial adherence to human epithelial cells increased by depletion of IgA as well as by depletion of IgM in serum, although the effect of IgA was not statistically significant (Fig 4B). Polymeric IgA specific to *V*. *cholerae* in serum may be an indirect indicator of blocked bacterial adhesion to intestinal cells. In fact, a small proportion of IgA may be directly secreted from gut-derived B cells in the blood [43], and up to 20% of serum IgA is found as dimer (the major form), trimer, or tetramer [44]. Furthermore, previous results showed that *V*. *cholerae* LPS-specific secretory IgA is increased in convalescent sera and intestinal fluids of cholera patients [7, 8]. In addition, serum IgA level correlates with protection against subsequent cholera infection [45]. It is important to note that secretory IgA does not induce complement-mediated bacterial lysis in a classical manner, and its main role is to prevent bacterial attachment in the gut [46, 47]. Thus, serum IgA may reflect the magnitude of secretory IgA in the gut, and its ability to block bacterial adhesion *in vitro* may be useful to assess intestinal colonization of *V*. *cholerae*.

IgM-mediated inhibition of bacterial adhesion seems to be associated with agglutination of *V*. *cholerae*. The colonization of *V*. *cholerae* to the epithelium of small intestine has been well described in several factors including TCP [48], GbpA [49], outer membrane proteins [50], and LPS [38]. Antibodies against colonization factors inhibited bacterial colonization, for example, anti-LPS antibodies inhibited bacterial adhesion to intestinal mucosa and induced agglutination of *V*. *cholerae* but such effect was not observed with antiserum devoid of anti-LPS antibodies [38]. Moreover, the previous study demonstrated that agglutination occurred in the both IgM and IgG-IgA fraction [14] which is consistent with our result that IgM-depleted or IgA-depleted sera could not inhibit bacterial adhesion whereas non-depleted serum did so. *V*. *cholerae* OSP or LPS-specific antibodies may arrest and/or agglutinate bacteria in the mucosal area and reduce opportunity to interact with intestinal epithelium [30, 51, 52].

The human colon carcinoma cell line, HT-29 cells, have been widely used for *in vitro* model for interaction between host and enteric pathogens due to the similarity of structure and their function [53, 54]. Especially, phenotypes of HT-29 cells after the differentiation are similar to those of small intestinal enterocytes in terms of their structure with brush-border microvilli and formation of tight junction, expression of the brush border-associated hydrolases, and the time course of the differentiation process [54]. In addition, HT-29 cells contain goblet cells secreting mucus that is abundant in the gastrointestinal epithelium and limits the colonizing bacteria in the gut [55]. Although adhesion of *V*. *cholerae* in the HT-29 cell may be useful *in vitro* tool to investigate the interaction with intestinal epithelium, there are some limitations to reflect *in vivo* phenomena of small intestine in respect to the different mucus layer. Goblet cells of small intestine secrete highly glycosylated mucin, MUC-2, and have higher viscosity and complex structure [56] while HT-29 cells contain a low proportion (<5%) of mucin-producing cells [55]. Furthermore, *V*. *cholerae* colonizes differently in the regions (proximal *vs*. distal) of small intestine [57].

Previous study reported that oral cholera vaccination preferentially increased intestinal IgG-secreting cells rather than IgM-secreting cells in the IgA-deficient patients [58]. Therefore, IgA-deficient patients might avoid vaccination with oral cholera vaccines because it may cause unexpected severe diseases, especially in patients with chronic inflammatory gut diseases in relation to IgG-mediated pathology such as inflammatory bowel disease and coeliac disease [59].

In conclusion, we demonstrated that anti-*V*. *cholerae* LPS IgM is highly correlated with serum vibriocidal activity and it could be a surrogate antibody isotype representing protective antibodies against *V*. *cholerae*.
